# External fixation to intramedullary nailing for femoral and tibial fractures: an eleven-year cohort study at a level I trauma center

**DOI:** 10.1007/s00590-025-04282-9

**Published:** 2025-05-27

**Authors:** Diego González-Morgado, Paula Fabado-Tortajada, Josep Nomdedéu, Jordi Teixidor-Serra, Jordi Tomàs-Hernández, Nayana Joshi-Jubert, Joan Minguell-Monyart, José Vicente Andrés-Peiró

**Affiliations:** 1 Department of Surgery, Universitat Autònoma de Barcelona, Barcelona, Spain; 2 Vall d’Hebron Hospital Universitari, Barcelona, Spain

**Keywords:** Intramedullary nailing, External fixation, Femur fracture, Tibia fracture, Pin tract infection, Fracture-related infection

## Abstract

**Purpose:**

To identify factors that contribute to the incidence of postoperative complications following staged treatment of femoral and tibial fractures with external fixation (EF) and intramedullary nailing (IMN).

**Methods:**

This retrospective cohort study involved patients with tibial and/or femoral fractures temporarily immobilized using EF, followed by IMN. Patient characteristics, types of injury, treatments, and outcomes were recorded. Primary outcomes were pin tract infection (PTI) and fracture-related infection (FRI).

**Results:**

The study had 103 patients involving 119 fractures: 73 tibial (61.3%) and 46 femoral (38.7%). Of these, 44.5% were open. 31.1% of the EFs were implanted by an orthopedic trauma (OT) specialist. In femoral fractures, OT specialists placed the pins a mean 78.2 mm from the fracture site, versus just 37.3 mm by non-OT surgeons (*p* < 0.01). This difference was not observed in the tibia. The average time of EF was 12.6 ± 7.8 days. PTI occurred in seven cases (5.9%), on average 14.9 ± 10.9 days after EF placement. FRI occurred in nine patients (7.6%): two in the femur (4.5%) and seven in the tibia (10.6%). All FRIs occurred in cases where the EF had been implanted by a surgeon without specialization in OT (*p* = 0.03). FRI was more frequent in patients with prior PTI than in those without (57.1% vs. 4.5%, respectively; *p* < 0.01).

**Conclusion:**

PTI was a risk factor for FRI after IMN of tibial and femoral fractures. Surgeon specialization in OT was a protective factor against FRI, probably related to pin placement further from the fracture site.

## Introduction

Intramedullary nailing (IMN) is the standard treatment for diaphyseal fractures of the tibia and femur [1, 2]. These fractures often occur in polytrauma patients with life-threatening injuries or severe soft tissue damage. In such cases, a damage control orthopedics approach with external fixation (EF) temporarily stabilizes the fractures while minimizing the burden of a more extensive surgical procedure [3, 4].

Infection following IMN is a devasting complication [5–7]. Although the use of previous EF has been shown to increase complication risk in several series [8–12], understanding of the factors that contribute to postoperative complications following this staged strategy is limited.

The primary aim of this study was to identify factors that contribute to postoperative complications following staged treatment with EF and IMN for femoral and tibial fractures. We hypothesized that the development of pin tract infection (PTI) and a reduced pin-to-fracture site distance would increase the rate of fracture-related infection (FRI). Additionally, we examined the impact of the treating surgeon’s training on treatment modalities and postoperative outcomes.

## Methods

This retrospective cohort study (level of evidence III) examined patients treated at a single level I trauma center between March 2011 and November 2022. We recruited all skeletally-mature patients (age ≥ 15 years) with femoral and/or tibial fractures who underwent temporary EF followed by IMN. We excluded cases involving indications for EF or IMN other than acute fracture fixation, pathological fractures, refractures, and any history of infection or neoplasms around the femur or tibia or their adjacent joints. To directly assess the impact of medullary cavity instrumentation by the pins, we also excluded patients who experienced a latency period between removal of the fixator and the nailing (“pin holidays”) and those in whom there was no overlap between the trajectories of the intramedullary implant and fixator pins. Institutional Review Board approval was obtained prior to study commencement (PR(AT)233/2022), and we adhered to the STROBE statement. Postoperative follow-up occurred over a one-year period.

All EFs were applied emergently as part of a damage control orthopedics approach, either local or systemic, by on-call orthopedic surgeons with varying levels of specialization in orthopedic trauma (OT). All definitive surgeries were conducted exclusively by OT surgeons. Open fracture management involved prompt antibiotic administration, debridement, and irrigation. When primary closure of wounds was infeasible, negative pressure wound therapy was utilized. The decision to use local antibiotics was made by attending surgeons. Open wound coverage was performed in collaboration with plastic surgeons. The EF pins underwent daily cleaning and antisepsis, per hospital guidelines. Conversion from EF to IMN was performed as soon as safely possible.

Fractures were classified using the AO/OTA system on emergency radiographs. The distance from the nearest EF pin to the closest fractured cortex on post-EF radiographs determined the pin-to-fracture distance. These same radiographs were used to measure the distance from the nearest pin to the center of rotation of the femoral head, the superior pole of the patella, or the center of the tibial eminence, and the talar articular surface, to assess the pin-to-hip, pin-to-knee, and pin-to-ankle distances, respectively. In radiographs following IMN, the degree of overlap of the nail with the EF’s pin trajectory was assessed. Each image was evaluated once by a single investigator blinded to outcomes.

We collected data on patient characteristics, their injuries, treatment provided, radiological evaluation, and outcomes. The primary outcome was the diagnosis of PTI and/or FRI based on clinical, radiological, and laboratory criteria [13, 14]. Secondary outcomes included any other postoperative complication.

Statistical analysis was performed using Stata/IC 14.2 (StataCorp, College Station, TX, USA). Continuous variables were summarized using means and standard deviations or medians and interquartile ranges (IQR), as appropriate. Categorical variables were represented using counts and percentages (%). To assess differences between continuous variables, the Student's t-test or Wilcoxon-Mann–Whitney rank-sum test were used, depending on data distribution. For categorical variables, differences were evaluated using the Pearson chi-square or Fisher's exact test, as appropriate. All *p*-values < 0.05 were considered statistically significant.

## Results

**Fig. 1:**
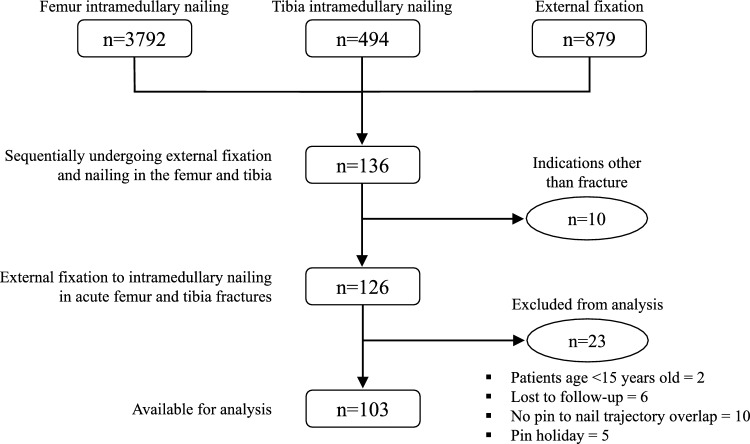
Flow diagram depicting patient selection process

A total of 103 patients of mean age 41.3 ± 18.8 years, with 119 fractures, were included (Fig. 1). Most patients were male (68%) and were predominantly involved in a traffic accident (60.2%), with a median Injury Severity Score (ISS) of 18 (IQR = 34). Among the 119 fractures, 73 were tibial (61.3%) and 46 femoral fractures (38.7%). Four patients had bilateral femur fractures, and two had bilateral tibia fractures. Nine patients had a floating knee, one being bilateral. We identified 53 open fractures (44.6%), more commonly in the tibia (54.8% tibia vs. 28.3% femur; *p* < 0.01). Gustilo IIIB fractures were prevalent in the tibia, accounting for 30% of open fractures in that bone (12 cases). Seventeen open fractures had a bone defect (32.1%), deemed critical or subcritical in eight cases [15]. Compartment syndrome requiring fasciotomy occurred in four cases (3.4%), three for the femur and one the tibia. Blisters were observed in 16 (21.9%) of the 73 tibial fractures, occurring in patients with a lower ISS (8.9 vs. 24.1; *p* < 0.01). Table 1 summarizes the study participants and their injuries.

**Table 1:** Patient’s characteristics, injuries, treatments, and outcomes

		Femur	Tibia	Overall
Patients and injuries	Sex (male/female)	73.9/26.1	64.4/35.6	68.1/31.9
	Age (years)	33.7 ± 13.3	44.0 ± 19.7	41.3 ± 18.8
	CCI^a^	0.2 ± 0.9	0.9 ± 1.8	0.6 ± 1.5
	ISS^b^	43.8 ± 18.2	20.7 ± 20.1	29.6 ± 22.4
	Open fractures	28.3	54.8	44.5
	*Gustilo classification*			
	I	–	11.0	6.7
	II	–	19.2	11.8
	IIIA	26.1	6.9	14.3
	IIIB	2.2	16.4	10.9
	IIIC	–	1.4	0.8
	*Bone defect classification *[13]			
	D1	4.4	9.6	7.6
	D2	6.5	2.7	4.2
	D3	–	4.1	2.5
	Compartment syndrome	6.5	1.4	3.4
Surgical treatment	Spanning frame	21.7	61.6	46.2
	Hip	8.7	–	3.4
	Knee	15.2	13.7	14.3
	Ankle	–	49.3	30.3
	Pin-to-fracture distance (mm)^c^	49.7 ± 41.5	71.8 ± 44.7	63.3 ± 44.6
	*Pin-to-joint distance* (mm)^d^			
	Hip	131.9 ± 57.6	–	131.9 ± 57.6
	Knee	77.0 ± 39.9	102.0 ± 41.7	92.6 ± 42.6
	Ankle	–	66.6 ± 50.6	66.6 ± 50.6
	Time of EF (days)^e^	12.3 ± 8.5	12.8 ± 7.3	12.6 ± 7.8
	Open reduction	37.0	12.3	21.9
	Pin-to-nail overlap^f^	3.5 ± 0.9	2.9 ± 1.1	3.1 ± 1
Outcomes	Pin-tract infection	4.4	6.9	5.9
	Fracture-related infection	4.4	9.6	7.6

Categorical variables are represented as percentages (%), and continuous variables as mean ± standard deviation

^a^Charlson’s Comorbidity Index

^b^Injury Severity Score

^c^Distance from the nearest EF pin to the closest fractured cortex on post-EF radiographs

^d^Distance from the nearest pin to the center of rotation of the femoral head, the superior pole of the patella or the center of the tibial eminence, and the talar articular surface on post-EF radiographs

^e^Days taken from external fixator application to intramedullary nailing

^f^Overlap of the nail with the EF’s pin trajectory on post-IMN radiographs

An orthopedic trauma (OT) specialist implanted 31.1% of the EFs. Roughly half (46.2%) the fixators were applied in a joint-spanning configuration. Spanning frames predominated in tibial over femoral fractures (61.6 vs. 21.7%; *p* < 0.01). In the tibia, spanning frames were used more frequently by OT surgeons (42.9% vs. 12.5% for non-OT specialists; *p* < 0.02). The average minimum pin-to-fracture distance was 63.3 ± 44.6 mm, being significantly longer for tibial than femoral fractures (71.8 vs. 49.7 mm; *p* < 0.01) and for spanning than sparing frames (92.9 vs. 37.8 mm; *p* < 0.01). For femoral fractures, OT specialists placed the pins an average of 78.2 mm from the fracture site, compared to 37.3 mm by non-OT surgeons (*p* < 0.01). Such a difference was not observed in the tibia. The mean minimum distance from the pins to the femoral head was 131.9 ± 57.6 mm: 92.6 ± 42.6 mm to the knee (superior pole of the patella or the tibial eminence), and 66.6 ± 50.6 mm to the ankle. This distance was consistently greater with spanning configurations than sparing frames: 200.3 vs. 114.7 mm at the hip, 115.1 vs. 74.3 mm at the knee, and 92.4 vs. 49.4 mm at the ankle, respectively (all *p* < 0.01). No cases of septic arthritis were identified; therefore, no association was found between this complication and the proximity of pins to joints. The average time of EF was 12.6 ± 7.8 days, though appreciably longer in patients undergoing a fasciotomy (33.5 vs. 11.9 days; *p* < 0.01). In 11 of 53 open fractures, local antibiotics were used, mainly in cases involving bone defects (29.4%) and Gustilo III open fractures (36.3%). PTI occurred in 5.9% of the EFs. No association was identified between this adverse event and patient characteristics, injury specifics, pin or frame configuration, surgeon’s profile or EF duration. PTIs appeared an average of 14.9 ± 10.9 days after EF. Diagnosis was primarily clinical, with microbiological confirmation in only three of seven cases (Table 2).

**Table 2:** Clinical and microbiological profiles of pin-tract and fracture-related infections

Sex, age^a^	ISS^b^	AO/OTA^c^	Gustilo^d^	PTI			FRI			
				EF to PTI onset^e^	Clinical criteria	Laboratory	IMN to FRI onset^g^	History and examination	Microbiology	Management^h^
M,19	75	32B		9	Painful, purulent drainage	CoNS^f^ + *E. coli*				
F, 35	48	31B		18	Persistent or increasing drainage					
M, 40	9	41 C	IIIA	34 (after EF removal)	Spreading erythema					
F, 71	9	42 C		8	Persistent or increasing drainage	*E. cloacae*	222	Fistula	*E. cloacae*	NR + RIA + AB IMN + flap
F, 88	9	44B		6	Local pain		106	Clinical signs + elevated serum markers	*E. cloacae*	NR + RIA
M, 30	9	42 C		24	Painful, purulent drainage	*E. cloacae*	33	Clinical signs + elevated serum markers	*E. cloacae*	NR + RIA + circular frame
M, 32	75	43B		5	Spreading erythema		312	Radiological signs (nonunion)	*C. acnes*	NR + RIA + AB IMN
F, 46	43	42 C	II				315	Clinical signs + elevated serum markers	CoNS^f^	NR + RIA
M, 41	9	42 C					299	Clinical signs + elevated serum markers	*E. cloacae*	NR + RIA + AB IMN
M, 34	75	32 A					217	Fistula	*E. coli*	DAIR
M, 16	18	32 A					62	Clinical signs + elevated serum markers	*C. acnes*	DAIR
M, 63	9	42 A	IIIB				241	Clinical signs + elevated serum markers	CoNS^f^	DAIR

^a^Shown as: M (male)/F (female), age (years)

^b^Injury Severity Score

^c^AO/OTA fracture classification

^d^Gustilo classification for open fractures. Empty for closed injuries

^e^Days from EF application to PTI onset

^f^Coagulase-negative Staphylococci

^g^Days from IMN to FRI onset

^h^NR: nail removal; RIA: reamer irrigator aspirator debridement; AB IMN: antibiotic-coated IMN, DAIR: debridement and implant retention

Diaphyseal fractures (AO/OTA 32 and 42) were the primary indication for IMN in both the femur (93.5%) and tibia (82.2%). Among these diaphyseal fractures, 44.2% in the femur and 34.2% in the tibia were classified as segmental fractures (AO/OTA 32 C and 42 C). Nineteen retrograde and 26 antegrade IMNs were implanted in the femur, 20 of the latter being cephalomedullary. In the tibia, 70 suprapatellar and three retrograde IMNs were implanted for tibiotalocalcaneal fusion. Open reduction was required for 21.9% of fractures before nail insertion, more often in the femur (37.0% vs. 12.3% for tibia; *p* < 0.01). On average, 3.1 ± 1.0 pinholes overlapped with the trajectory of the IMN. The number of pins that overlapped the nail was significantly greater in the femur than tibia (3.5 vs. 2.9; *p* < 0.01) and for non-spanning than spanning frames (3.9 vs. 2.2; *p* < 0.01). All Gustilo IIIB fractures were covered with a free flap, with 11 undergoing"fix and flap"within a median of nine days (IQR = 6). In the remaining three, coverage was initially performed on EF, followed by conversion to IMN. The three critical bone defects, one in the femur and two in the tibia, were managed using an induced membrane technique.

We identified nine FRIs (7.6% of fractures): two in the femur (4.5%) and seven in the tibia (10.6%) (Table 2). All FRIs occurred in cases where the EF had been implanted by a non-OT surgeon (p = 0.03). FRI was more frequent in patients with prior PTI than in those without (57.1% vs. 4.5%, respectively; *p* < 0.01). In two FRIs with a history of PTI, the microbiological diagnosis was the same for each (*E. cloacae*). In the tibia, the minimum distance from the pins to fracture site was shorter in cases with an FRI (42 mm vs. 74.9 mm); however, this difference was not statistically significant (p = 0.07). The presence of blisters was associated with an increased incidence of tibial FRI (57.1% vs. 18.2%, respectively; *p* < 0.04). Neither the presence of an open fracture nor the time to flap influenced the risk of FRI. FRIs appeared a median of 222 days after nailing (IQR = 221.5). In six of nine cases, treatment required removing the IMN. We found two aseptic non-unions caused by mechanical problems, one in the femur and one in the tibia, which needed revision of fixation surgery. All remaining fractures healed within the study period. Joint stiffness in the knee developed in seven cases, with four involving femoral and three tibial fractures.

## Discussion

In this study of 119 femoral and tibial fractures sequentially treated with EF and IMN, we observed a 5.9% incidence of PTI and 7.6% incidence of FRI, potentially highlighting a relationship between these complications. Notably, the use of EF by a surgeon specialized in orthopedic trauma (OT) was identified as a protective factor against infection.

Fractures of the tibia and femur that occur in polytrauma patients often require a damage control orthopedics strategy with EF followed by IMN [3, 4]. The timing of conversion is influenced by several factors, including soft tissue condition, need for further debridement, EF pin condition, and the patient's physiological state [16]. Delaying definitive surgery to the 6 th to 8 th day has been shown to decrease the inflammatory response relative to earlier intervention [3]. In our study, the time to IMN conversion was longer than 6–8 days and quite variable, likely due to both significant trauma-related systemic involvement and local conditions in the affected extremity, as seen in the high rates of open fractures and soft tissue damage. This highlights the heterogeneity of these patients and the need for an individualized approach when determining the optimal timing for definitive surgery.

Local infection attributed to EF is the most commonly-argued downside of this staged treatment [17]. In most studies, PTI rates were between 5 and 25% [8, 17–19], consistent with our findings. Although the risk factors for PTI are not fully understood, it is recommended that surgeons carefully select their pin sites and avoid areas with soft tissue damage [19–22]. Prolonged EF duration can also contribute to PTI, as extended exposure increases the risk of bacterial colonization [19–22]. For instance, PTI and postoperative infection rates rise significantly when EF remains in place for more than 2 weeks [8, 9, 17, 23]. Our low PTI rate was likely due to early conversion to IMN, within two weeks in most cases, and strict adherence to a standardized pin-care protocol. Although proper pin care is likely crucial, there is currently no consensus on best practices [21, 22, 24, 25]. While additional preventative options for PTI, including pin coating, exist [19], further measures to prevent PTI remain necessary.

The incidence of FRI following IMN of the tibia and femur has ranged widely, from 1 to 23% [5–7], being higher after conversion from EF (6–67%) [8–12, 18], thereby aligning with our findings. Several factors contribute to FRI, including fracture type, open fractures, prior use of EF, and the need for soft tissue reconstruction [5, 6, 9, 10]. Similarly, we found an association between FRI and PTI, as well as soft tissue damage. Conversely, we failed to identify any association with the duration of EF, open fractures, or other injury characteristics.

Several strategies have been proposed to reduce FRI after EF [9, 12, 17, 18, 23–33]. While early conversion to IMN as a single procedure is generally recommended [9, 12, 26], some authors advocate for a two-stage conversion or “pin-holiday” approach [29, 31–33], especially when a PTI is present [10, 11, 18, 28]. Additional preventative measures include pin track debridement, soft tissue excision, over-drilling of bony pin holes, and administering antibiotics when PTI first becomes evident [9, 17, 27]. While we generally avoided two-stage conversions, we applied these additional measures during IMN conversion when PTI was suspected or when conversion occurred more than two weeks after external EF. Other authors have recommended performing systematic cultures of the IMN reaming and subsequent use of postoperative antibiotics based on final culture results [12]. However, their high number of contaminated samples raises concerns about the reliability of this practice [12]. We found that microbiological organism remained the same in two patients who developed FRI after PTI, agreeing with Bunzel et al.’s findings [9]. This highlights the risk of bacteria migrating from EF pin sites into the intramedullary canal, potentially causing deep postoperative infections, emphasizing the need for careful patient selection when using EF in fractures likely to need IMN later.

While the principles for pin placement in EF for fracture stability are well established, research on how pin placement affects consolidation and infection is limited [16, 34]. Although it is generally recommended that surgeons avoid placing pins in areas of soft-tissue damage or fracture hematoma, how the distance from the pin to fracture site impacts outcomes has not been thoroughly investigated. We found that patients with tibial fractures who developed FRI had pins placed closer to the fracture site. Although this finding did not reach statistical significance, this may merely have been because of inadequate statistical power. Proximity to the injury or fracture hematoma can increase the risk of PTI due to higher contamination levels in these areas, particularly with open fractures. Additionally, pins placed too close to the fracture site may disrupt local soft tissue and periosteal blood supply, which are crucial for bone healing and infection resistance [35].

Previous studies on ankle and tibial plateau fractures have revealed that surgeon specialization does not influence EF placement or postoperative outcomes [36, 37]. Conversely, in our study, the use of EF by a surgeon specialized in OT was protective against infection, likely due to more refined surgical techniques and greater adherence to maintaining safe distances between pins and fracture sites, which was probably facilitated by the more frequent use of joint-spanning frames. Notably, all FRIs occurred in patients managed by non-OT surgeons. These findings emphasize the importance of advanced training in EF for orthopedic surgeons to ensure optimal temporary fracture stability while minimizing the risk of infectious complications.

We acknowledge several limitations of this study, including its retrospective design, the heterogeneity of the injuries (encompassing both femoral and tibial fractures, open and closed), and the diversity of treatment modalities employed. Despite these limitations, the study offers clinically relevant insights by demonstrating a strong association between prior pin tract infection and fracture-related infection, identifying surgeon specialization as a protective factor, and introducing pin-to-fracture distance as a potentially modifiable surgical variable. These findings are supported by an integrated analysis of radiological, microbiological, and surgical data from a large real-world cohort, enhancing the robustness and clinical value of our results.

In conclusion, prior pin-tract infection appears to significantly increase the risk of fracture-related infection after intramedullary nailing of tibial and femoral fractures. Conversely, specialization in orthopedic trauma may be protective against infections, likely due to more strategic pin-site selection.

## Data Availability

No datasets were generated or analysed during the current study.
